# Epitaxial growth of high-quality GaN with a high growth rate at low temperatures by radical-enhanced metalorganic chemical vapor deposition

**DOI:** 10.1038/s41598-024-61501-9

**Published:** 2024-05-13

**Authors:** Arun Kumar Dhasiyan, Frank Wilson Amalraj, Swathy Jayaprasad, Naohiro Shimizu, Osamu Oda, Kenji Ishikawa, Masaru Hori

**Affiliations:** Center for Low-Temperature Plasma Science (cLPS), Nagoya University, Furo-cho, Chikusa, Nagoya 464-8603 Japan

**Keywords:** Compound semiconductor, Radical enhanced metalorganic chemical vapor deposition, Gallium nitride, Growth rate, GaN/GaN power device, Materials science, Materials for devices, Electronic devices

## Abstract

Using our recently developed radical-enhanced metalorganic chemical vapor deposition (REMOCVD) technique, we have grown gallium nitride (GaN) on bulk GaN and GaN on Si templates. Three features make up this system: (1) applying very high-frequency power (60 MHz) to increase the plasma density; (2) introducing H_2_ and N_2_ gas in the plasma discharge region to produce active NH_x_ radical species in addition to nitrogen radicals; and (3) supplying radicals under remote plasma arrangement with a Faraday cage to suppress charged ions and photons. Using this new REMOCVD system, it was found that high-quality crystals can be grown at lower temperatures than that of MOCVD but the disadvantage was that the growth rate was smaller as 0.2–0.8 μm/h than that by MOCVD. In the present work, we have used a pBN inner shield to prevent the deactivation of radicals to increase the growth rate. The growth conditions such as the plasma power, trimethylgallium (TMG) source flow rate, N_2_ + H_2_ gas mixture flow rate, and the ratio of N_2_/H_2_ were optimized and it was found that the growth rate could be increased up to 3.4 μm/h with remarkably high crystalline quality comparable to that of MOCVD. The XRD-FWHM of GaN grown on the GaN/Si template and the bulk GaN substrate were 977 arcsec and 72 arcsec respectively. This work may be very promising to achieve high-power GaN/GaN devices.

## Introduction

Gallium nitride (GaN) finds numerous applications in opto- and electronic devices, especially high-electron-mobility transistors (HEMT)^1^, laser diodes (LDs)^2,3^, and light-emitting diodes (LEDs)^2,4^. The growth of GaN is widely investigated using hydride vapor phase epitaxy (HVPE), metalorganic chemical vapor deposition (MOCVD) and molecular beam epitaxy (MBE). However, GaN is commercially manufactured as thin epitaxial layers on various substrates mainly by MOCVD^5^.

Growth occurs layer by layer in the molecular-beam epitaxy (MBE) method. High-purity GaN can be grown in the ultra-high vacuum (UHV) environment, with a low operating pressure of about 10^–5^ Torr. MBE can only be used in lab-scale research because it is unfeasible for large-scale industrial applications because of its high operating costs and challenging growth process scalability.

The primary technique for producing GaN films commercially on a variety of substrates, including sapphire, SiC, and Si, is MOCVD. This process requires a temperature of more than 1000 °C, for crystal growth and it consumes a large amount of raw materials, such as TMG and ammonia (NH_3_). Ammonia gas is decomposed thermally by pyrolysis to N radicals and NH_x_ radical species. The decomposition efficiency is however very low so that the large consumption of ammonia (NH_3_) is necessary, several thousand times more than that is needed. Most of the ammonia is detoxicated and exhausted without usage. Therefore, ammonia cost takes a large part (around 30–50%) of the total production cost. It is also similar to metalorganic (MO) materials whose consumption efficiency is low so they also increase the cost.

The GaN grown on large-diameter wafers face significant challenges due to wafer breakage and bowing, as high temperatures are required in MOCVD for the thermal excitation of ammonia gas. This is due to the difference in the thermal expansion coefficient and the lattice constant between the substrate and the epitaxially grown layers.

To solve the above disadvantages of MOCVD, It is desired to develop a novel growth technology that enables low-temperature growth with low consumption of raw materials. The low-pressure MOCVD to produce reactive nitrogen species (N-radicals) from plasma for nitride growth has attracted significant interest. This is because it enables GaN growth at lower temperatures, thereby reducing wafer bowing caused by the difference in thermal expansion coefficients between the substrate and grown layers. Additionally, the fact that it can lower the production cost of GaN crystal growth without the use of NH_3_ gas. Over the last twenty years, numerous studies have documented the formation of GaN films by the plasma-enhanced MOCVD (PEMOCVD) process, which involves TMG reacting with N-radicals produced by plasma under various excitation frequencies^6–25^. These previous studies on PEMOCVD have been performed by plasma generation with radio frequency (RF, 13.56 MHz), radio frequency inductively coupled plasma (RF-ICP)^10,13–15^, helicon source (13.56 MHz with magnet)^11,12^, microwave (2.45 GHz without magnet)^6–9,17–19^ and electron cyclotron resonance (ECR) (2.45 GHz with magnet)^16,20–23^ but were far from the industrial application because high-quality GaN films have not been achieved with the comparable quality as those grown by the conventional MOCVD. As seen from the perspective of plasma generation, higher radical densities can result from higher plasma densities. Higher radical densities can be produced using generation sources like helicon and ECR due to their efficient electric power coupling with plasma. However, because of the confined and uneven nature of the plasma creation, they are not suitable for affordable large-scale mass production.

Therefore, we have developed a novel REMOCVD method^26,27^, in which nitrogen and hydrogen gas can be exited in plasma, and nitrides can be grown by the reaction between radicals and metal–organic (MO) gas. Presently, industrial equipment for large-diameter substrates of 150–300 mm is required for applications to power devices and low-cost LEDs^28–30^. We are therefore developing a 300 mm diameter REMOCVD system^31^.

We have grown GaN, AlN, InN and AlInN layers by this REMOCVD method^26,27,31–64^ and found that it is very effective to grow these nitrides at much lower temperatures than those of the MOCVD method without using any ammonia gas. It can be proved that the crystal quality is close to that of MOCVD even though the growth temperature is much lower^27^.

The REMOCVD method has however a disadvantage in that the growth rate is about 0.2–0.8 μm/h, lower than that of MOCVD whose growth rate is higher than 2 μm/h. For REMCOVD to be applied industrially, it is essential to increase the growth rate. Theoretically, we believe that the growth temperature itself is not a key factor for increasing the growth rate since III metal atoms will diffuse on the surface very quickly because the surface diffusion coefficient is very large due to their low melting points^65,66^. The key factor will be the radical density and energy which will meet the III metal supply amount and give the appropriate V/III ratio. We therefore tried to increase the TMG gas flow rate under high radical density conditions but the growth rate was not increased as we predicted as explained in “Effect of plasma power” section. From this result, we speculate the following mechanisms: (1) most radicals may be deactivated because their sticking coefficient to the stainless chamber wall is very large^67^; (2) generated radicals are not focused towards the substrate surface because the chamber volume is too large and most of the radicals diffuse to all directions; and (3) because of the large chamber volume, the electron temperature is too low to have sufficient energy to generate atomic nitrogen radicals. We therefore tried to use a pBN inner shield in the process chamber to protect radicals from colliding with the stainless chamber wall to be deactivated and to focus the radicals towards the substrate surface (scattering of radicals is avoided) and to generate more atomic nitrogen radicals. By this new procedure, we could increase the growth rate of GaN up to 3.4 μm/h even at a low growth temperature of 800 °C without using ammonia gas. In this paper, we will report the increase of the growth rate of GaN by optimizing the growth conditions, using the pBN inner shield.

Vertical GaN power switching technology is expected to be utilized in next-generation medium to high-voltage power converters due to the high breakdown voltage and low on-resistance compared to Si and SiC-based devices^68^. The properties of nitrides are no longer dominated by defects introduced by heteroepitaxial growth, allowing the recent realization of several fundamental vertical power devices, including diodes with edge termination, trench MOSFETs, and CAVETs. For GaN vertical power devices with a large current and a high breakdown voltage, high-quality crystals are required. This work may be very promising to achieve high-power GaN/GaN devices.

## Experimental

**Figure 1:**
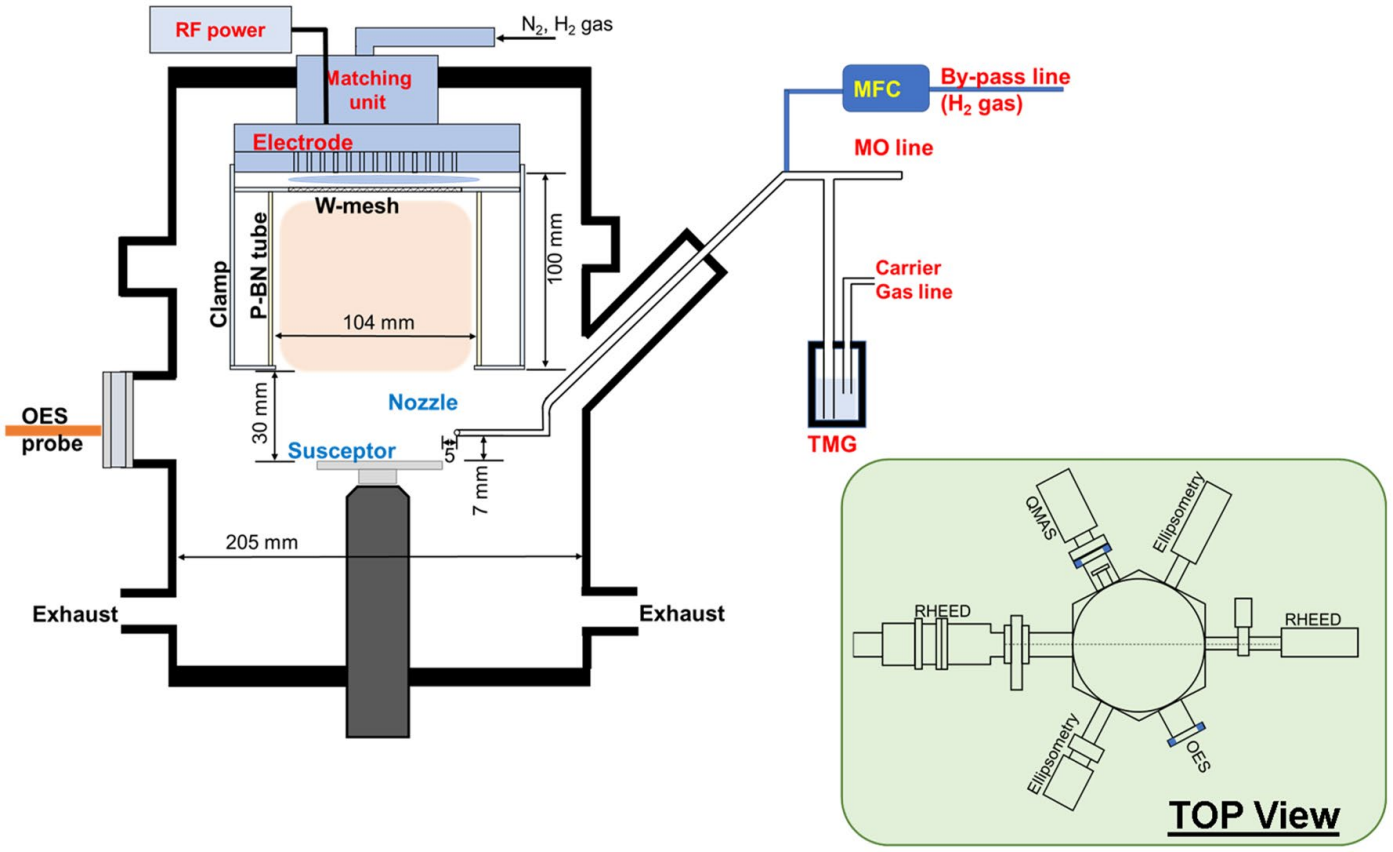
Experimental setup of REMOCVD with a pBN inner shield and in-situ RHEED, QMAS and spectroscopic ellipsometer systems.

The REMOCVD system was described in detail elsewhere^26,27^ and Fig. 1 shows the experimental arrangement of the REMOCVD system for the present work. The system consists of a chamber having a capacitively coupled plasma source and a sample stage. The exhaust lines are connected to a turbo molecular drug pump and the background pressure was around 10^–4^ Pa. An N_2_ and H_2_ mixture gas with a flow rate of 75–2250 sccm for N_2_ and 25–1500 sccm for H_2_ with a total flow rate of 100–3000 sccm was introduced through a showerhead of the top electrode^26^. The pressure was maintained at 100–300 Pa by the automatic pressure controller equipped with the exhaust line. Plasma was generated by applying 200–800 W of a very high frequency (VHF, 100 MHz) power to the top electrode, distanced approximately 150 mm from the substrate surface. Ammonia radical species (NH_x_) and reactive nitrogen species (N radicals) including excited N_2_ and N atoms are generated in the plasma discharge region. Plasma was separated by a metal mesh to prevent charged particles and emitted light generated in the plasma discharge region to reach the substrate^26^. Substrates were placed on the sample stage with a susceptor made of carbon and coated by SiC. A hoe-type gas exit with five pipes was set to a level of approximately 7 mm distance from the substrate surface^26^. TMG gas was supplied from a bottle controlled at − 12 °C to + 5 °C with and without H_2_ carrier gas (0–20 sccm) for studying the effect of TMG flow rate. The TMG flow rate ranging from 1.2 to 3.4 sccm was controlled by the needle valve.

Using an optical fiber probe positioned at the plasma discharge region, distanced 25 mm above the sample stage, radicals and reactive species generated by plasma were identified through a spectral analysis on the elemental atomic emission lines detected by HR2000 high-resolution optical emission spectrometer (OES) (Ocean Optics, Inc.).

Bulk GaN substrates (Na flux method; NGK corp.) with the size of 10 × 10 × 0.35 mm and GaN/Si templates (about 1 μm-GaN grown on (111)Si with an AlN buffer layer of about 1 μm; Dowa Corp.) with the size of 10 × 10 × 0.625 mm^3^ were used for this study. Before the growth, a wet cleaning procedure was carried out. The substrate was ultrasonically cleaned in an acetone bath, and in an isopropanol bath for 5 min and was then rinsed with deionized (DI) water. The substrate was then dipped in 5% hydrofluoric acid for 5 min and rinsed with deionized (DI) water^32^. Finally, the substrate was dried using an N_2_ gas and loaded into the chamber for subsequent in-situ cleaning. Inside the chamber, N_2_/H_2_ plasma cleaning of the substrate was carried out at 200 W for 20 min.

Subsequently, the substrate temperature was heated and maintained at 800 °C for the main epitaxial growth of GaN and the crystal growth was performed mainly for 120 min. This growth temperature of 800 °C is significantly low compared with the growth temperature of approximately 1000–1050 °C for the conventional MOCVD using ammonia gas^26^. In-situ reflection high-energy electron diffraction (RHEED) system (STAIB Instruments; DP-RHEED30) was installed in the REMOCVD system and was used to observe the crystallinity of grown GaN layers. Spectroscopic ellipsometry (SE) (J. A. Woollam Co., Inc M-2000F) and quadrupole mass spectrometer (QMAS) (Qulee-ULVAC, QCM-BGM-202) were also installed to observe the gas species.

After crystal growth, the surface morphology and the cleaved cross-section were observed by scanning electron microscopy (SEM; Hitachi Hi-Technologies SU-8230). Crystalline quality was evaluated by double crystal X-ray diffraction (XRD) using a high-resolution diffractometer HRXRD (9 kW Rigaku Smartlab diffractometer, Rigaku Co. Ltd.) with the arrangement of θ-2θ for measuring X-ray Bragg diffraction angle and of ω with a constant θ for X-ray rocking curve measurement.

## Results

### Optical emission spectra (OES)

**Figure 2:**
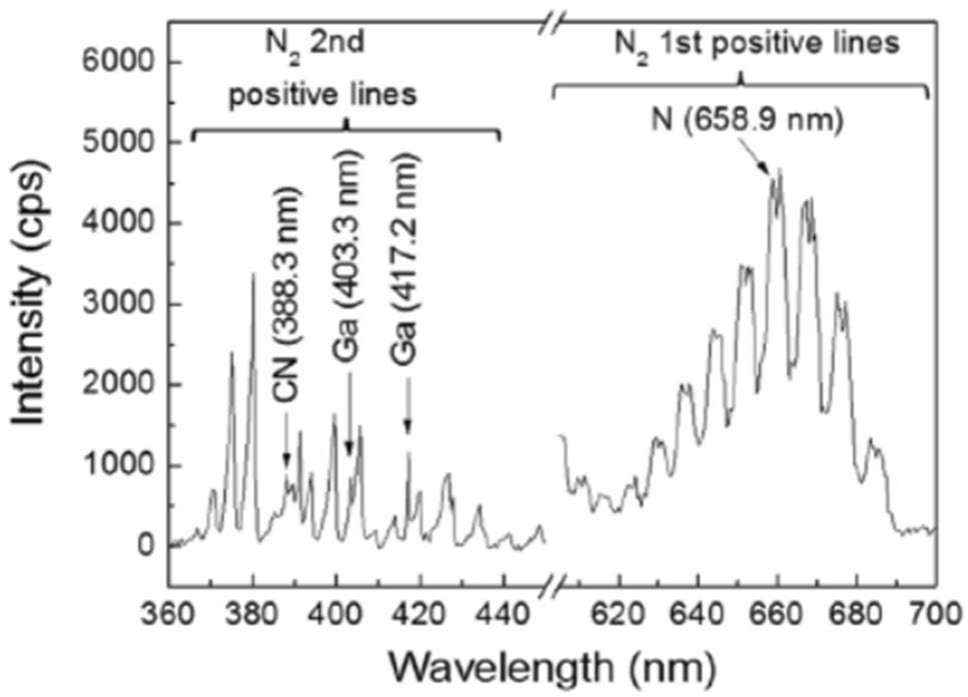
Typical OES in the REMOCVD system. Reproduced with permission^26^. Copyright 2014, Elsevier Publishing.

The plasma provides various reactive nitrogen species such as grounded N atom (^4^S°), metastable N atom (^2^D°, ^2^P°) and electronically excited N_2_ molecules (A^3^Σ_u_^+^), created by electron-collisions to the ground state of N_2_ (X^1^Σ_g_^+^) as shown in Fig. 2. By the introduction of H_2_ into the N_2_ plasma, ammonia molecules are also created in the N_2_ and H_2_ plasma^69–76^. To date, few studies have been carried out to optimize growth conditions for high-quality GaN films in the N_2_/H_2_ PEMOCVD^8,10,13,15,19^. We have however used a mixture gas of H_2_ and N_2_ to optimize the growth conditions^27^. The advantage of this gas mixture will be the formation of ammonia radical species (NH_x_).

**Figure 3:**
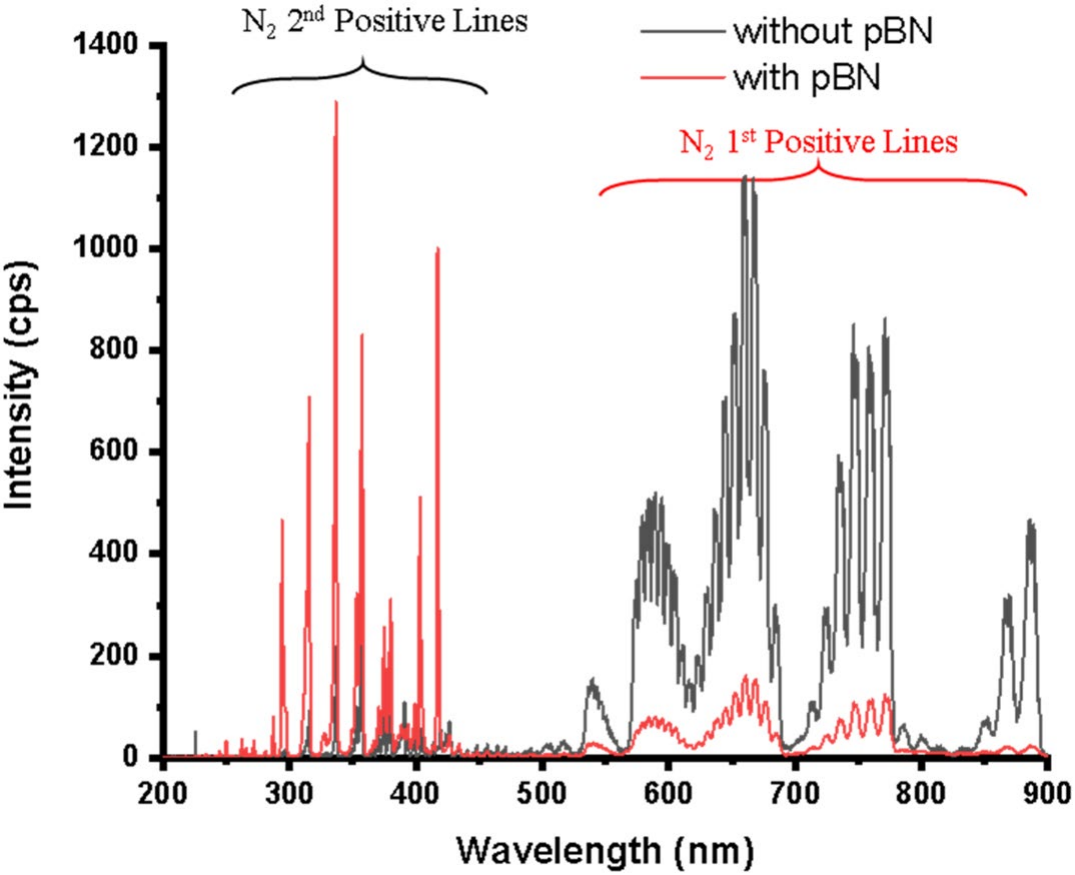
OES spectra of N_2_ plasma with and without pBN inner shield for the N_2_ flow rate of 750 sccm with the RF power of 600 W.

Figure 3 shows a typical optical emission spectrum of N_2_ plasma with and without a pBN inner shield for our REMOCVD system. In the case of nitrogen species, we observe two bands, the N_2_ 1st positive system and the N_2_ 2nd positive lines and atomic N. The 2nd positive series of N_2_ (C^3^Π_u_–B^3^Π_g_) and 1st positive series of N_2_ (B^3^Π_g_–A^3^Σ_u_^+^) were observed in the wavelength range of 300–450 nm and 500–900 nm, respectively^22^. Atomic nitrogen transition peaks, such as 4p^2^So–3s^2^P, are visible at roughly 491.5 and 493.5 nm, even though they are not strong enough, the related species could be involved in the formation of GaN crystals. The first positive ion N_2_ and the second positive ion N_2_ have shorter lifetimes^23^, and they may contribute to the growth of the GaN crystal. The 2nd positive N_2_ line intensity of N_2_ plasma with the pBN inner shield increases compared with the case without the pBN inner shield. This may be because the electron temperature was increased due to the plasma concentrated inside the pBN inner shield and the atomic nitrogen density was increased. Higher electron temperature means higher energy which induces large excitations in the higher energy 2nd N_2_ positive lines. Since the electron temperature increases because of the pBN inner shield, the low energy 1st N_2_ positive line intensity decreases.

**Figure 4:**
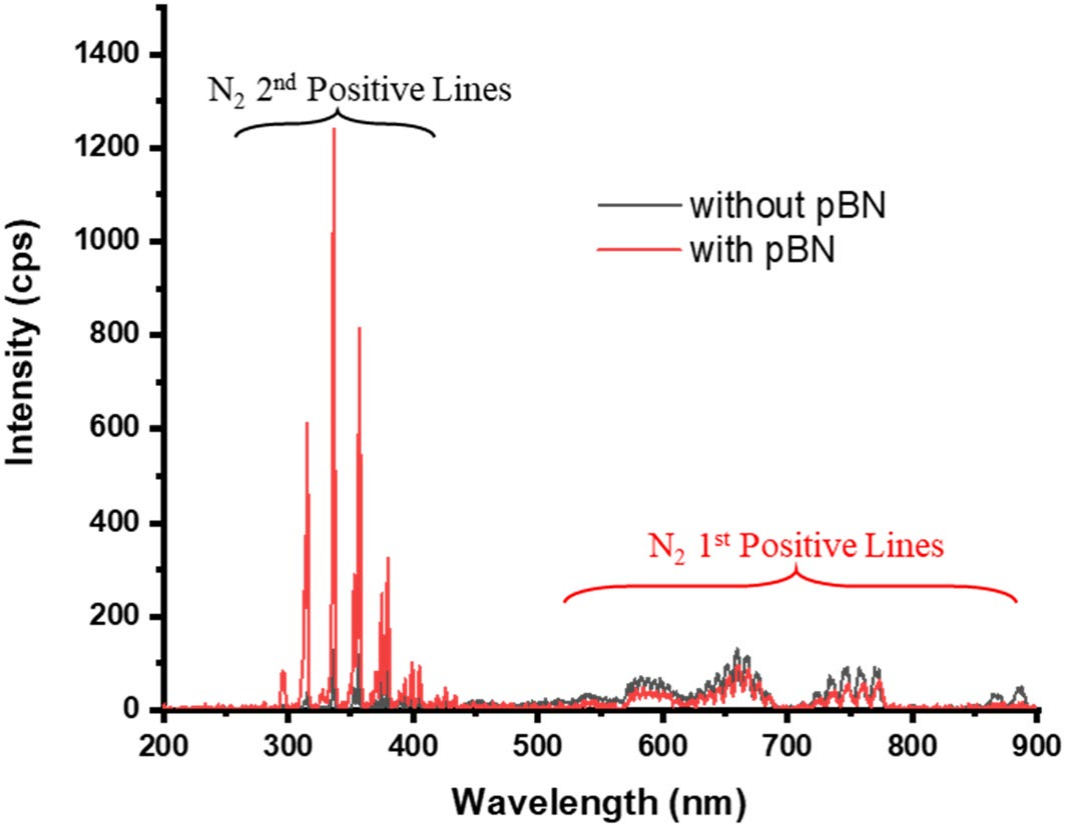
OES spectra of N_2_/H_2_ plasma with and without pBN inner shield for the flow rate of N_2_/H_2_: 750/250 sccm with the plasma power of 600 W.

Figure 4 shows a typical optical emission spectrum of N_2_/H_2_ plasma with and without a pBN inner shield. For the case without the pBN inner shield, N_2_ 1st positive lines are significantly decreased with the addition of H_2_ source gas and no significant decrease was observed for N_2_ 2nd positive lines. For the case with pBN inner shield, there is no significant changes were observed on N_2_ 1st positive lines and N_2_ 2nd positive lines.

In the observed emission spectra (Figs. 3, 4), we could identify all major lines which were observed by the discharge of an H_2_ and N_2_ mixture gas. All lines arising from the N_2_ system including transitions among A^3^Σ_u_^+^, B^3^Π_g_ and C^3^Π_u_ levels are identified as vibrational–rotational quantization based on quantum dynamics treatment^67,73^. The peak at around 336 nm seems to be due to emissions from NH molecules, transitions between levels A^3^Π and X^3^Σ^−^^72^ but is difficult to make sure because the peak overlaps with a line of the N_2_ 2nd positive series. Since NH molecules are reactive, we further need to analyze them from the viewpoint of their transport to the surface and their role in the GaN film growth. The detection of NH molecules is noteworthy for understanding the mechanism of GaN growth in our newly developed system.

CN peaks are commonly observed in PEMOCVD^13^ but were weak in our case as shown in Fig. 3, probably because CN species are reduced by H radicals or they were hardly generated because of our remote plasma arrangement. CN species are not desired since they contaminate grown GaN films with carbon.

The N_2_ and H_2_ plasma was diagnosed in our earlier investigation on the construction of the radical source using quadruple mass spectroscopy (QMS) and vacuum ultraviolet absorption spectroscopy (VUVAS)^77–79^. We found that the chemical species such as ammonia, H atom, and N atom are formed in the plasma at the flow ratio of 750 sccm for N_2_ and 250 sccm for H_2_. However, the current experiment differs greatly from the prior one in that N_2_ and H_2_ create stable NH_3_ molecules due to a significantly higher gas pressure in the radical source. Since the pressure in the plasma discharge region is in the range of 100 to 300 Pa, it is speculated that reactive NH or/and NH_2_ species exist in the plasma discharge region of our system and reach the sample surface. Based on initial findings, we think that these metastable ammonia species could be crucial to the growth of GaN. Most of them react with TMG, which is absorbed at the surface of the growing crystals, meaning that very little NH_3_ gas is released from the system. When we checked the exhausted gas with an ammonia detection shield, it was detected only in a small amount of 0.2 vol%, using a gas-detecting tube.

**Figure 5:**
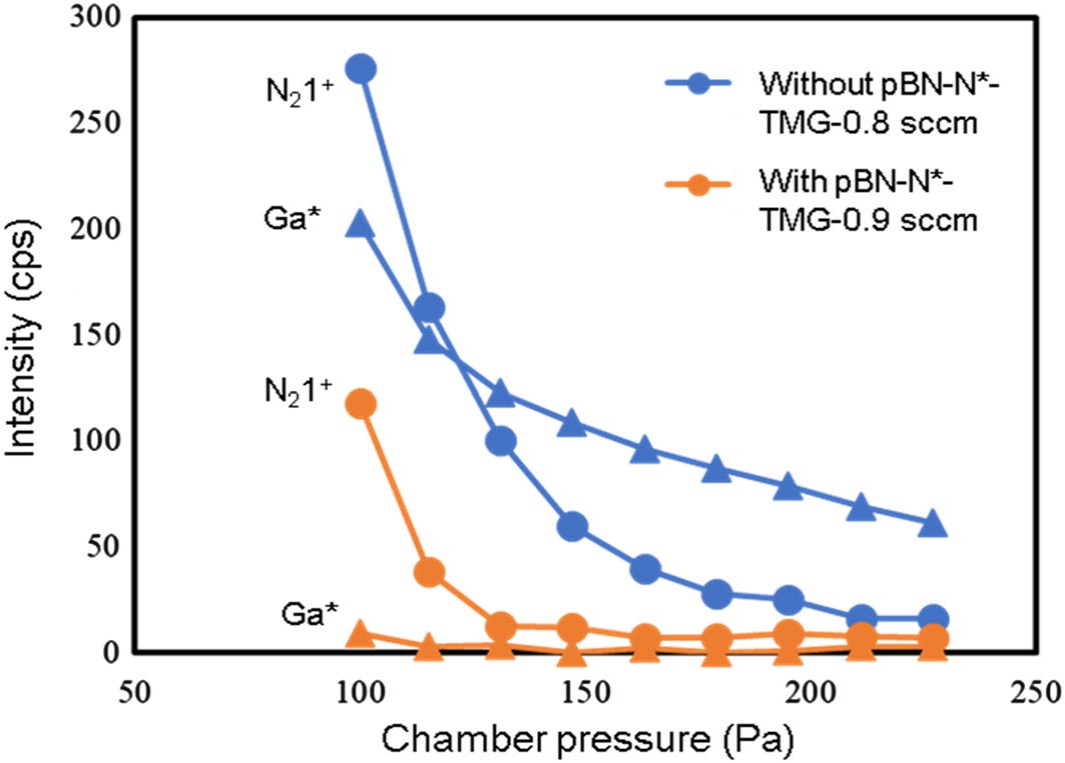
OES of the Ga* and N_2_ 1st positive line with and without pBN inner shield as a function of the chamber pressure.

**Figure 6:**
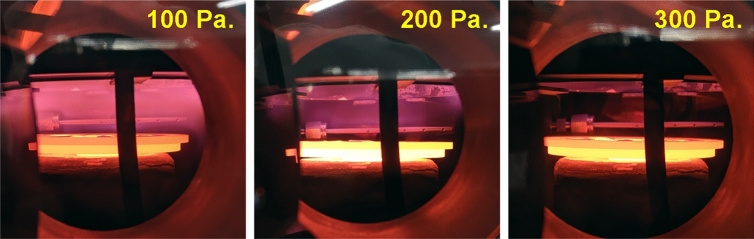
Plasma emission as a function of chamber pressure.

Figure 5 shows the OES intensity of Ga* and N_2_* 1st positive line with and without pBN inner shield as a function of the chamber pressure. For both with and without a pBN inner shield, the OES intensity decreases exponentially as a function of chamber pressure. It can be known that under 100 Pa, the optical emissions are very strong and they become small under higher chamber pressure due to the collisions of radicals because of the less mean free path of the radicals. Figure 6 shows the photograph of the emission near the sample holder for different chamber pressures with the pBN inner shield. For 100 Pa chamber pressure, we could see a sheath layer around the sample holder. It shows that the plasma is so strong, that it may promote the etching of GaN rather than the growth of GaN. The intensity of the sheath layer decreases with the increase of chamber pressure and it becomes invisible when the chamber pressure is 300 Pa. From the viewpoint of crystal growth the higher chamber pressure is desired since the plasma region does not reach the sample and will give less damage but for studying the optical emissions of various radicals, the lower chamber pressure is desired.

### Growth conditions

#### Effect of plasma power

As reported till now^26,32–51^, we found the optimized condition of GaN without the pBN inner shield is as follows;

- Plasma power; 600 W;
- N_2_/H_2_ gas flow rate: N_2_; 750 sccm, H_2_; 250 sccm;
- Chamber pressure: 100 Pa;
- TMG flow rate: 0.1 sccm (TMG bottle temperature; − 12 °C);
- Growth temperature: 800 °C;
- Growth rate: 0.4 μm/h.

However, the growth rate was limited to about 0.4 μm/h. To increase the growth rate, we have increased the TMG flow rate to increase the Ga supply and examined the growth rate under the assumption that sufficient nitrogen radicals are supplied with a high plasma power of about 600 W.

**Figure 7:**
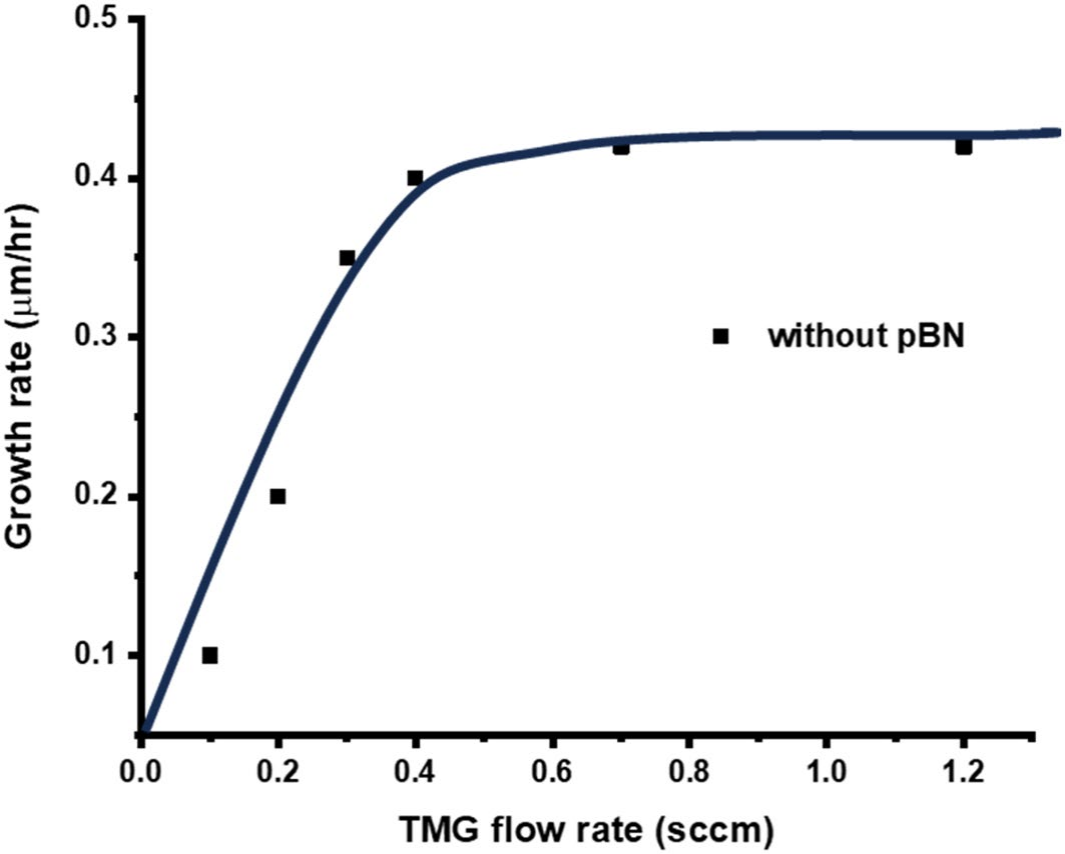
The growth rate as a function of the TMG flow rate without the pBN inner shield.

Figure 7 shows the result of the growth rate as a function of the TMG flow rate but it was found that the growth rate was saturated at about 0.4 μm/h. This result was disappointing since theoretically speaking the growth rate should be increased with increasing the TMG flow rate when the density of nitrogen radical species is sufficient. We therefore concluded that the most of nitrogen radical species will be deactivated with their collision with the stainless wall. We, therefore, attached a pBN inner shield in the chamber in a way that nitrogen radicals will not be deactivated by their collision with the stainless steel wall. PBN is known that it has less sticking coefficient for radicals^67^.

**Figure 8:**
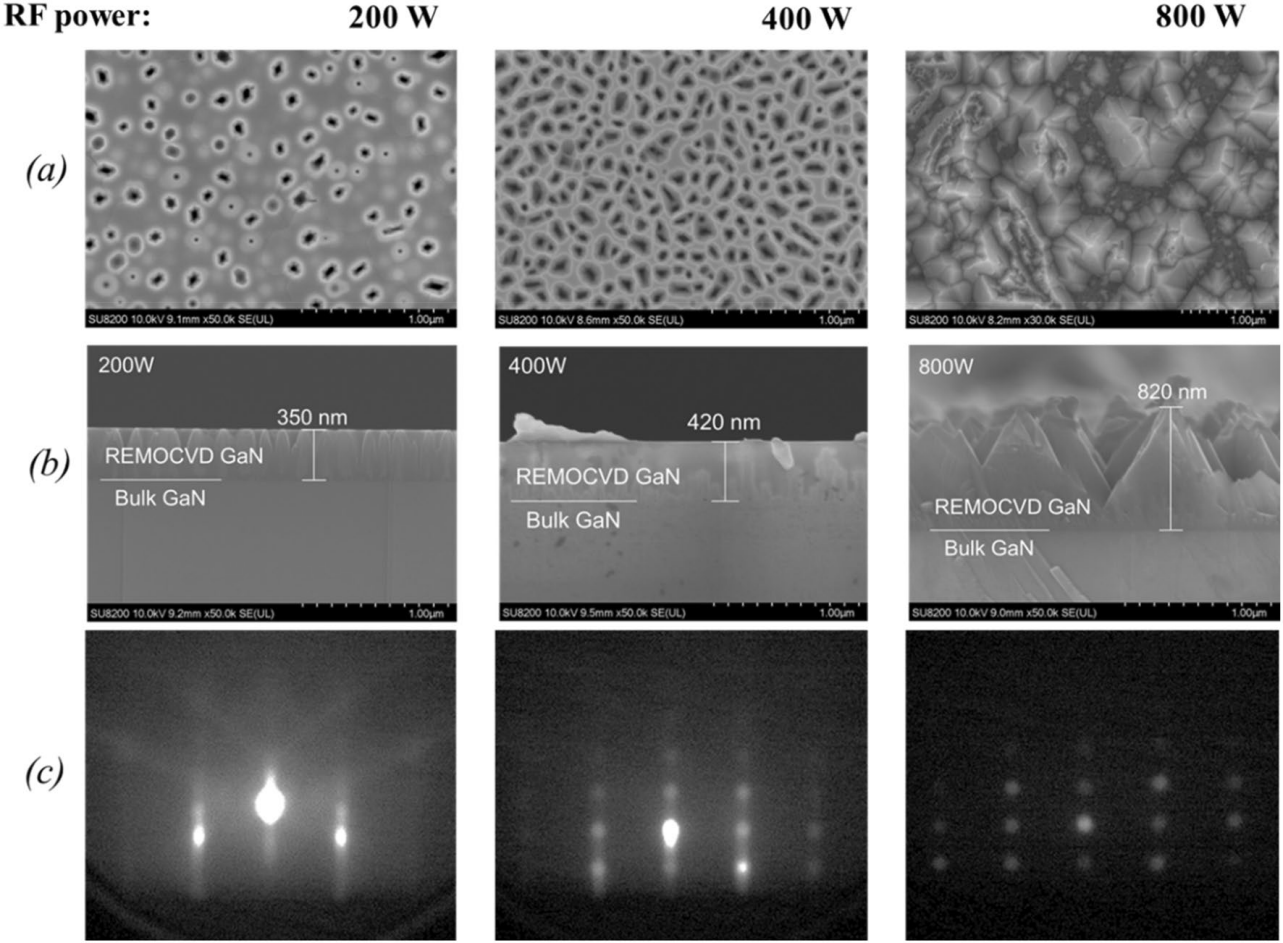
Growth of GaN on bulk GaN substrates as a function of the plasma power. (**a**) Surface morphology, (**b**) cross-section by SEM and (**c**) RHEED patterns. Growth conditions; 800 °C, 120 min, 300 Pa, N_2_/H_2_ = 750/250 sccm, TMG − 12 °C.

Figure 8 shows the growth experiment results by changing the plasma power. As predicted, it was found that flat GaN layers can be obtained with plasma power as low as 200 W. It means that by using the pBN inner shield, sufficient nitrogen radicals can be provided even at 200 W, while in the case of without the pBN inner shield, the plasma power as high as 600W is necessary to grow flat GaN. In the case of the growth without the pBN inner shield, it was impossible to grow GaN with such a low plasma power. Figure 8 also shows that by increasing the plasma power to 400 W and 800 W, three-dimensional GaN could be grown with a high growth rate. We can see that the surface is rough, dominated by islands. This is a typical pattern expected for systems with slow adatom diffusion. Since there is too much N compared to Ga adatoms, Ga adatoms don’t have enough time to diffuse over the surface and find the proper site for growth. The incoming N quickly reacts with Ga adatoms and starts to build islands and this leads to 3D growth. These results imply that it is promising to increase the growth rate of GaN by increasing the TMG flow rate with the higher plasma power.

#### Effect of the TMG gas flow rate

##### Increase in the TMG bottle temperature

**Figure 9:**
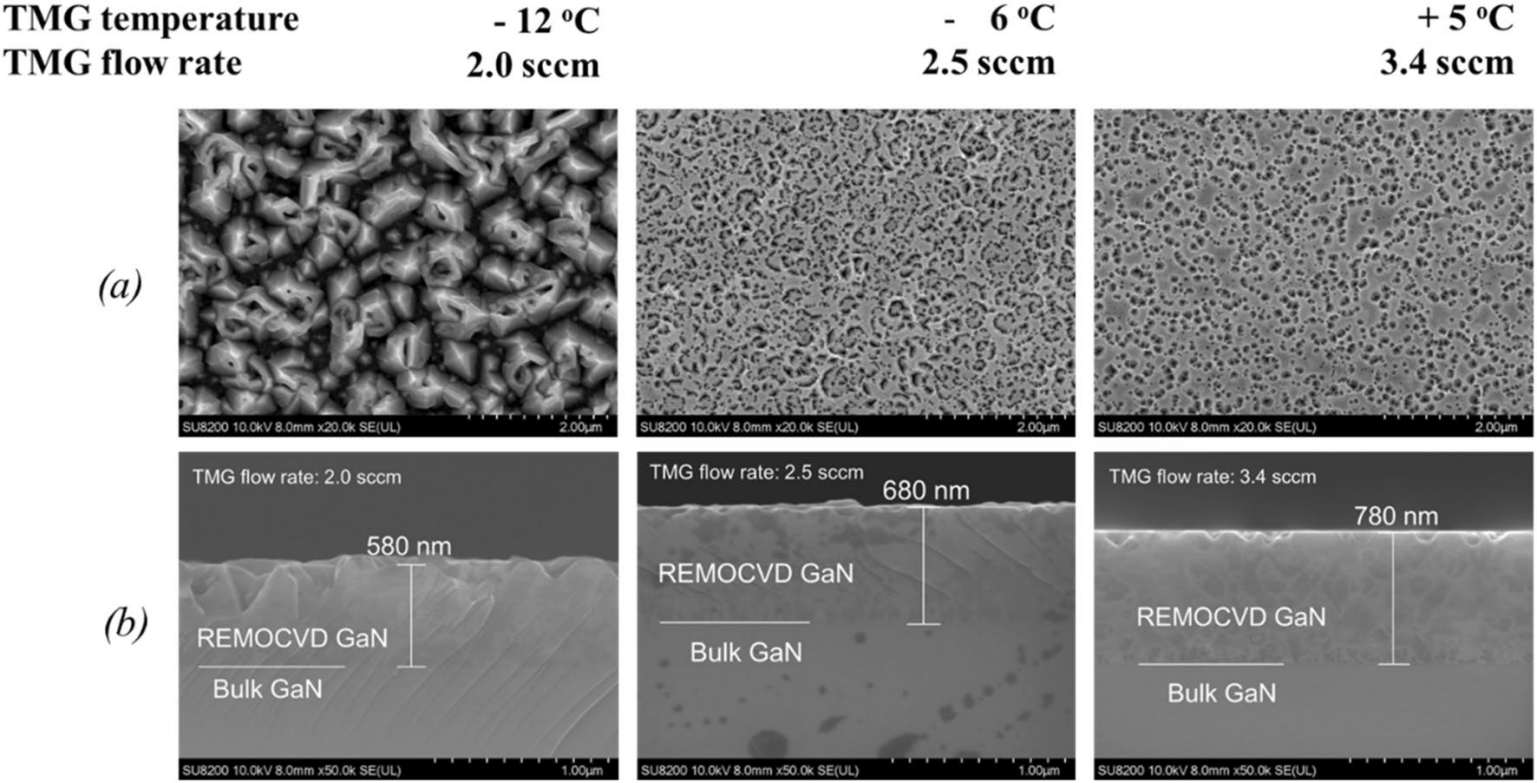
Growth of GaN on bulk GaN substrates as a function of TMG bottle temperature. (**a**) Surface morphology and (**b**) cross-section by SEM. Growth conditions; 800 °C, 120 min, 110 Pa, N_2_/H_2_ = 750/250 sccm.

**Figure 10:**
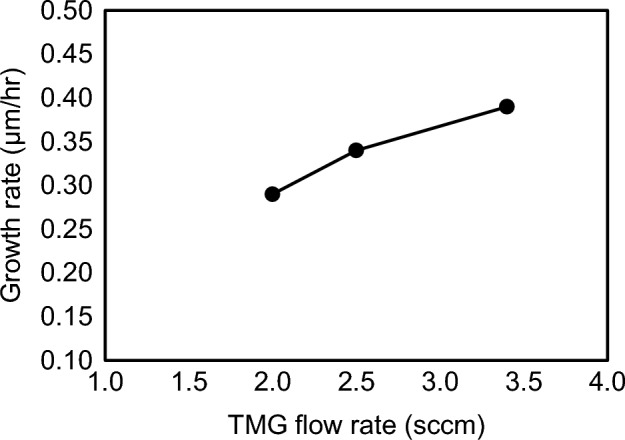
Growth rate of GaN as a function of the TMG bottle temperature. Growth conditions; 800 °C, 120 min, 110 Pa, N_2_/H_2_ = 750/250 sccm.

Since it was found that GaN could be well grown even at 200 W, we started to study to increase the amount of Ga to optimize the V/III ratio at 600 W where the density of nitrogen radical species will be larger. First, the TMG flow rate was increased by increasing the TMG gas bottle temperature from − 12 °C (TMG flow rate; 2.0 sccm) to 5 °C (TMG flow rate; 3.4 sccm). As expected, the growth rate was increased from 290 to 390 nm/h as shown in Figs. 9 and 10. The surface morphology became better by increasing the TMG flow rate. With the low TMG flow rate, the morphology was three-dimensional because it was under N-rich conditions.

##### Increase of the H_2_ the carrier gas flow rate

**Figure 11:**
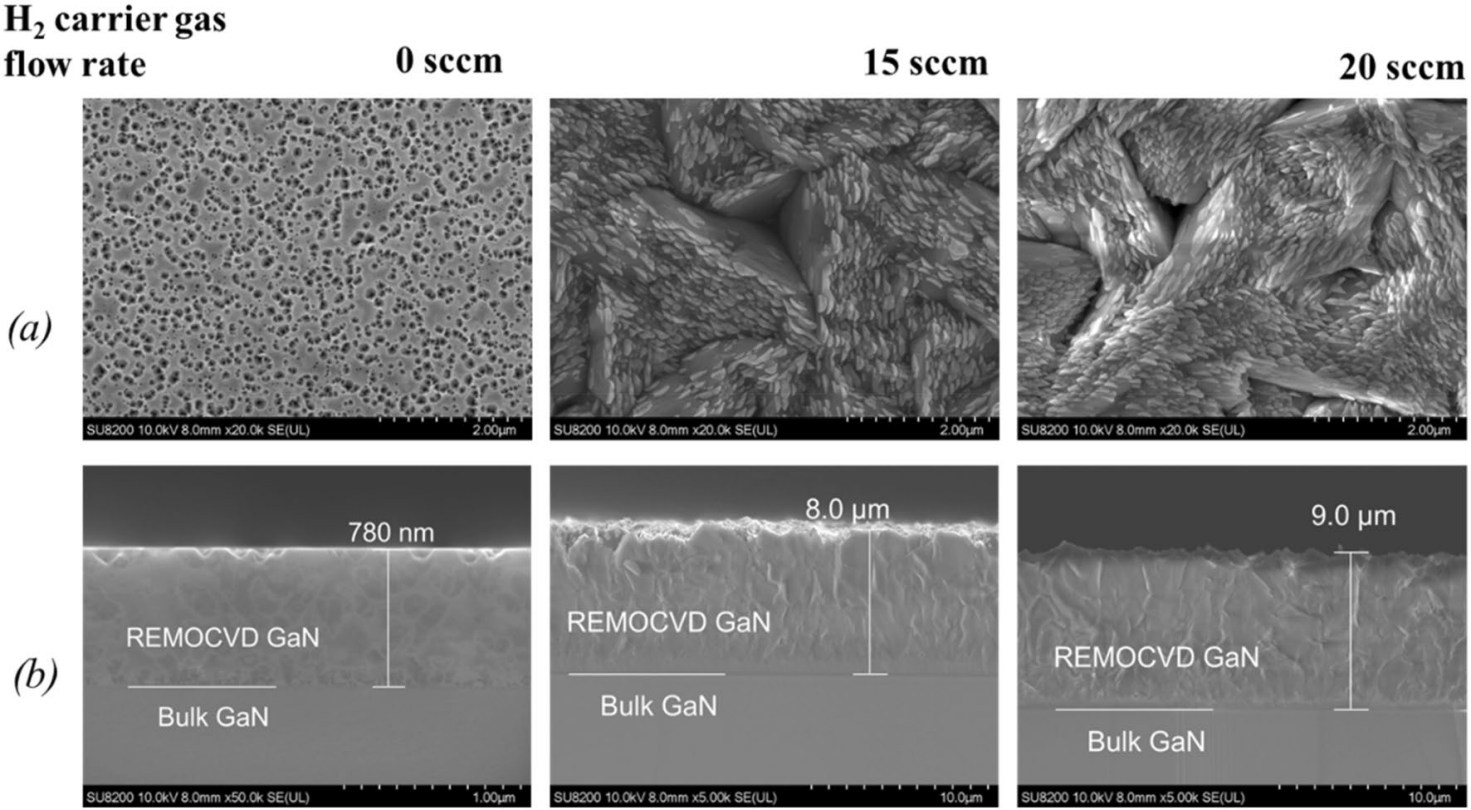
The growth of GaN on bulk GaN substrates as a function of H_2_ carrier gas flow rate for TMG gas supply. (**a**) Surface morphology and (**b**) cross-section by SEM. Growth conditions; 800 °C, 120 min, 110 Pa, N_2_/H_2_ = 750/250 sccm, TMG + 5 °C.

**Figure 12:**
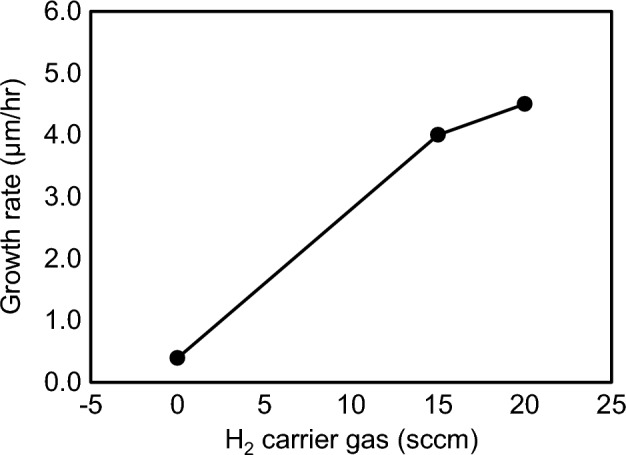
The growth rate of GaN as a function of the H_2_ carrier gas for the TMG gas supply. Growth conditions; 800 °C, 120 min, 110 Pa, N_2_/H_2_ = 750/250 sccm, TMG + 5 °C.

We then increased the TMG flow rate by adding the H_2_ carrier gas from 0 to 20 sccm. We used hydrogen as a carrier gas to optimize the growth conditions because the TMG decomposes at low temperatures in the H_2_ atmosphere compared to N_2_. The effect was transcendently impressive as shown in Figs. 11 and 12. The growth rate was increased from 390 nm/h to 4.0 μm/h for 15 sccm of H_2_ carrier gas and to 4.5 μm/h for 20 sccm of H_2_ carrier gas. The growth rate was so much increased as we expected but the surface morphology was not satisfactory.

#### Effect of N_2_/H_2_ flow ratio

##### Change of the N_2_/H_2_ flow ratio with a constant N_2_/H_2_ total gas flow rate of 1000 sccm with the plasma power of 200 W

**Figure 13:**
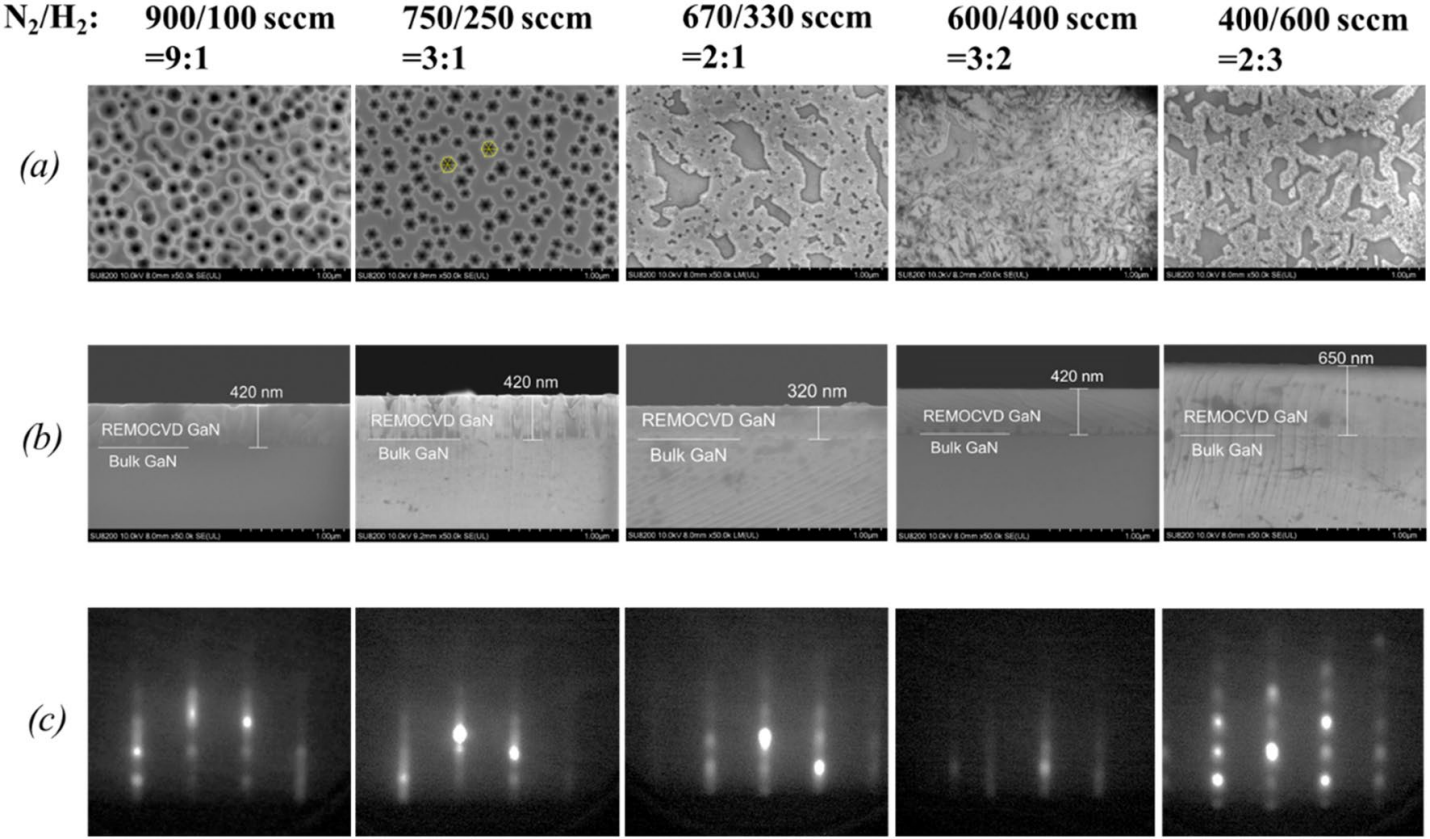
Growth of GaN on bulk GaN substrates as a function of the N_2_/H_2_ ratio with a constant total flow rate of 1000 sccm and with the plasma power of 200 W. (**a**) Surface morphology, (**b**) cross-section by SEM and (c) RHEED patterns. Growth conditions; 800 °C, 120 min, 200 W, 110 Pa, N_2_/H_2_ = 750/250 sccm, TMG − 12 °C/1.2 sccm.

**Figure 14:**
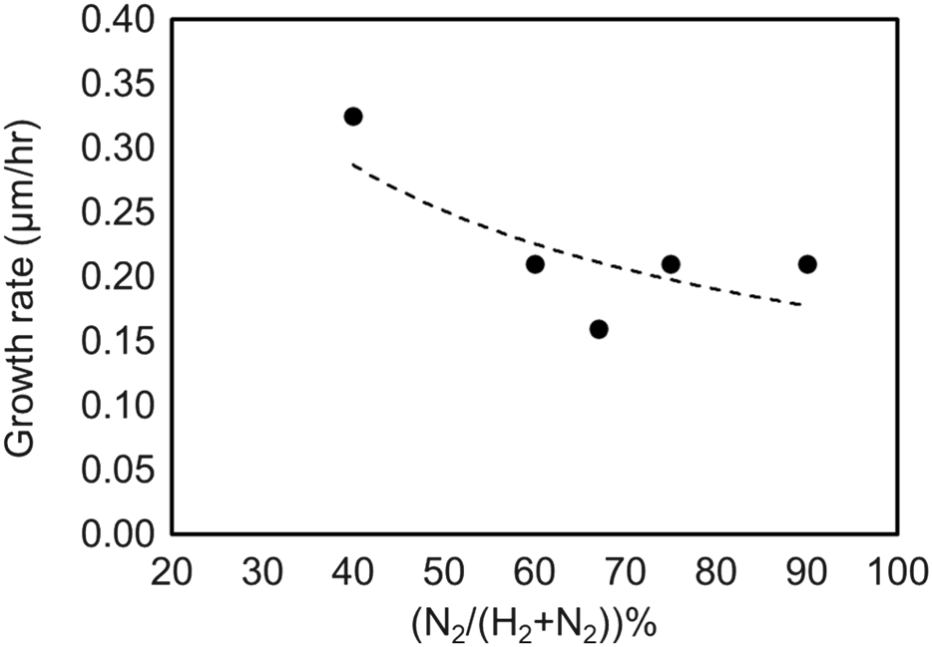
Growth rate of GaN on bulk GaN substrates as a function of the H_2_ percentage per (H_2_ + N_2_) with a constant total flow rate of 1000 sccm and with the plasma power of 200 W. Growth conditions; 800 °C, 120 min, 200 W, 110 Pa, N_2_/H_2_ = 750/250 sccm, TMG − 12 °C/1.2 sccm.

To check the N_2_/H_2_ flow ratio, we first studied its effect with a constant N_2_/H_2_ total gas flow rate of 1000 sccm as shown in Figs. 13 and 14. It was found that the surface morphology becomes better by decreasing the N_2_/H_2_ ratio as also shown in our previous report^27^. This may be due to the increase of NH radicals with increasing the H_2_ ratio. It is known that the pit formation is prevented by decreasing the N_2_/H_2_ ratio and the RHEED patterns with streak diffraction lines could be observed at the N_2_/H_2_ ratio of 3:2.

##### Change of the N_2_/H_2_ flow ratio with a constant H_2_ flow rate of 250 sccm with the plasma power of 600 W

**Figure 15:**
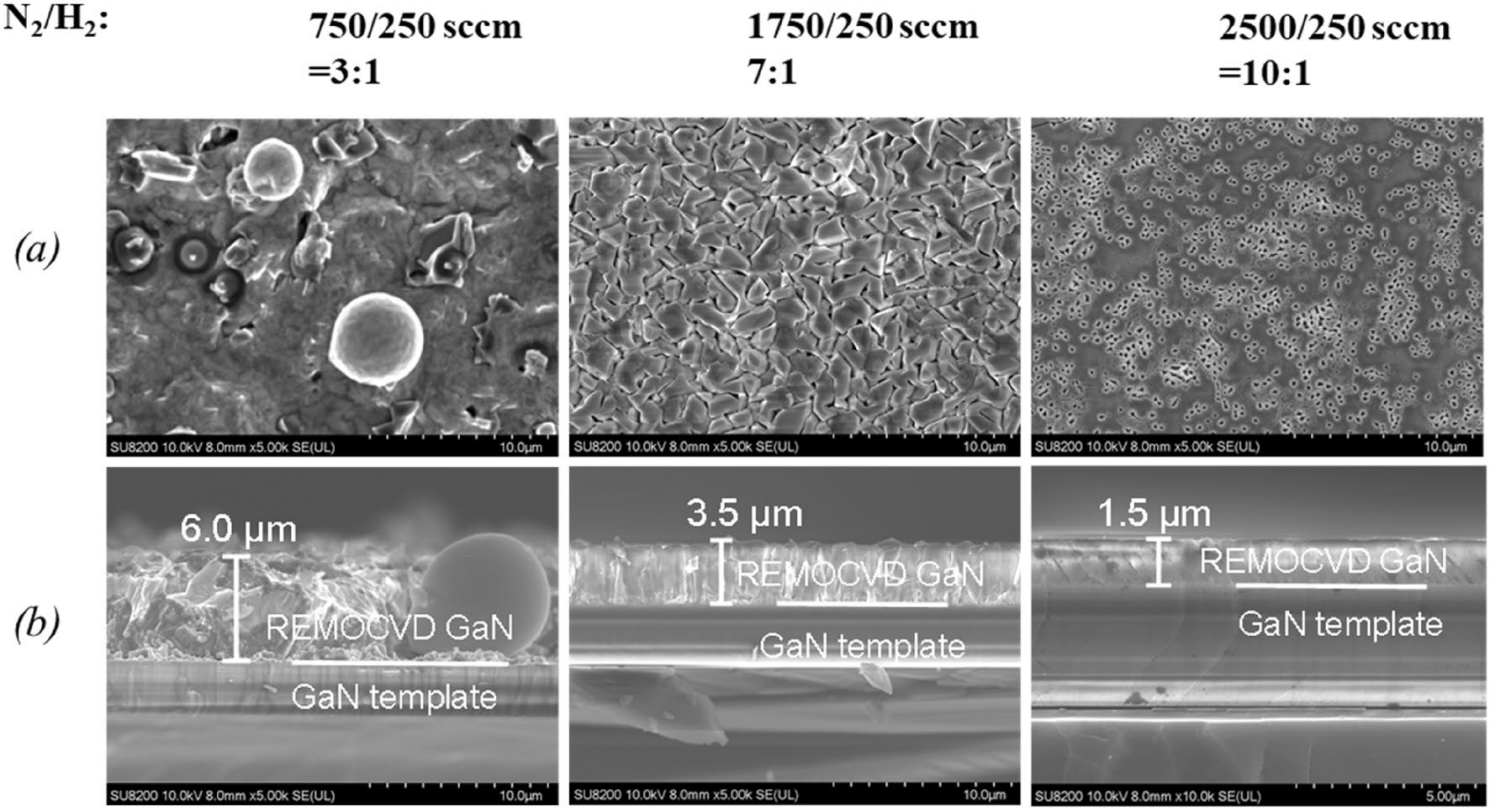
Growth of GaN on GaN templates as a function of the N_2_/H_2_ total flow rate with the same H_2_ gas flow rate of 250 sccm. (**a**) Surface morphology and (**b**) cross-section by SEM. Growth conditions; 800 °C, 120 min, 600 W, 300 Pa, N_2_/H_2_ = 750/250 sccm, TMG + 5 °C/H_2_ carrier gas 20 sccm.

We have studied the effect of the total flow rate of N_2_/H_2_ mixture gas on the growth morphology and the growth rate. As shown in Fig. 15, it was found that by increasing the total flow rate, the morphology becomes flatter, and the thickness becomes thinner. This result shows that by increasing the total flow rate of the N_2_/H_2_ mixture gas, the nitrogen radical density becomes larger and the appropriate V/III ratio was achieved.

##### Change of the flow ratio with a constant N_2_/H_2_ total gas flow rate of 2000 sccm with the plasma power of 600 W

**Figure 16:**
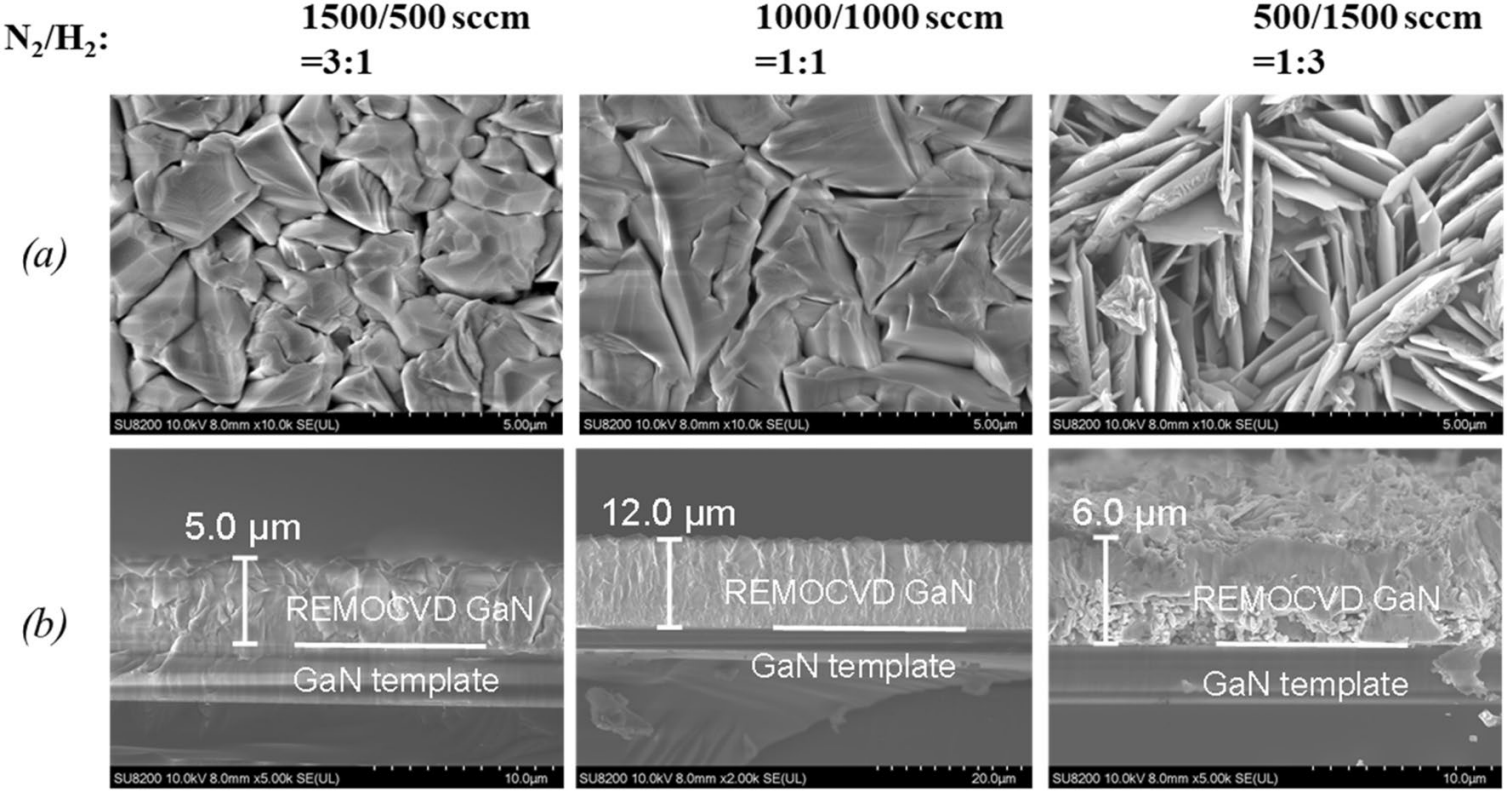
The growth of GaN grown on GaN templates by changing the N_2_/H_2_ ratio from 3:1 to 1:3 with a constant N_2_/H_2_ total gas flow rate of 2000 sccm. (**a**) Surface morphology and (**b**) cross-section by SEM. Growth conditions; 800 °C, 120 min, 600 W, 300 Pa, TMG + 5 °C/H_2_ carrier gas 20 sccm.

Since we found that the growth rate can be increased with increasing the N_2_/H_2_ total flow rate, we have examined the effect of the N_2_/H_2_ flow ratio in the case of the N_2_/H_2_ total flow rate of 2000 sccm as shown in Fig. 16. It was found that the growth rate is very dependent on the N_2_/H_2_ flow ratio as shown in Fig. 16 and that the growth rate can be increased up to 6 μm/h in the case of N_2_/H_2_ ratio is 1:1, two times more compared with the case of N_2_/H_2_ ratio is 3:1 or 1:3.

#### Effect of N_2_/H_2_ total gas flow rate

##### Change of the N_2_/H_2_ total gas flow rate with the same N_2_/H_2_ gas flow ratio of 3:1

**Figure 17:**
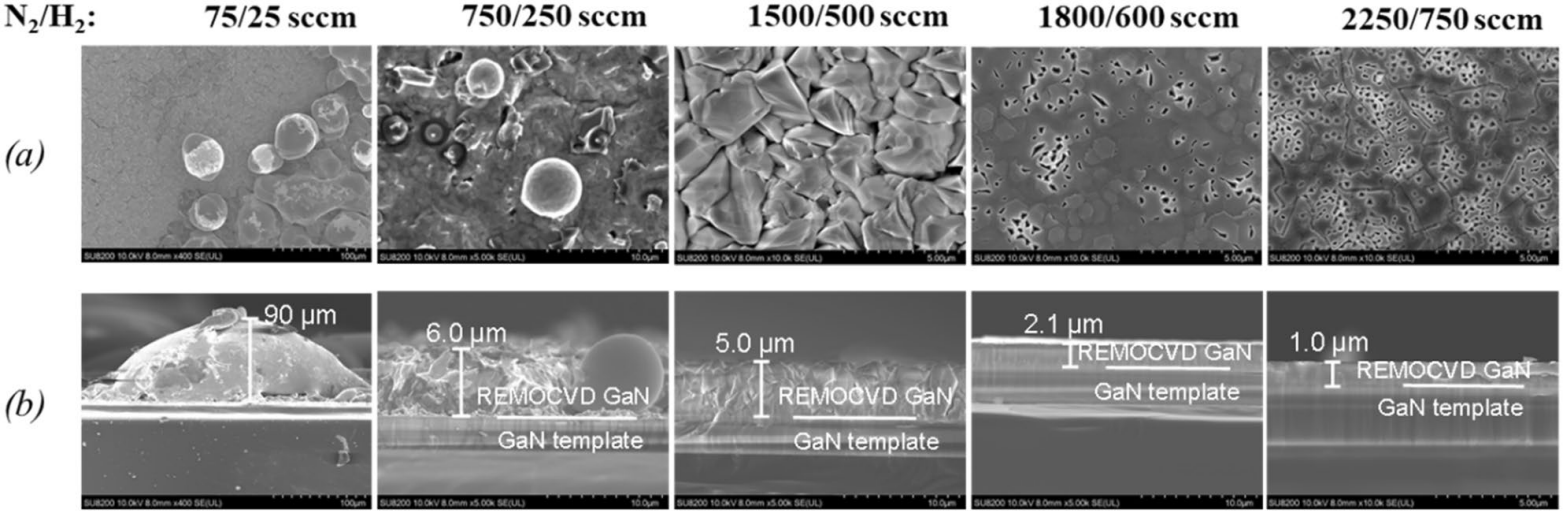
Growth of GaN grown on GaN templates as a function of the N_2_/H_2_ total gas flow rate with the same N_2_/H_2_ gas flow ratio of 3:1. (**a**) Surface morphology and (**b**) cross-section by SEM. Growth conditions; 800 °C, 120 min, 600 W, 300 Pa, TMG + 5 °C/H_2_ carrier gas 20 sccm.

**Figure 18:**
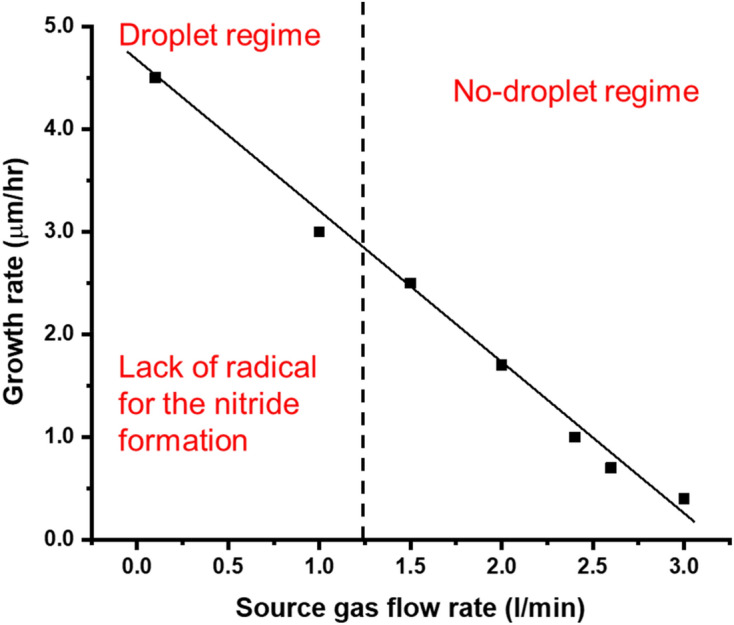
The growth rate as a function of the N_2_/H_2_ total gas flow rate with the same N_2_/H_2_ gas flow. Growth conditions; 800 °C, 120 min, 600 W, 300 Pa, TMG + 5 °C/H_2_ carrier gas 20 sccm.

The total gas flow rate of N_2_/H_2_ source gas from the showerhead was increased from 100 to 3000 sccm with the same N_2_/H_2_ gas ratio of 3:1. It was found that in the slow total gas flow of 100 sccm, there were Ga droplets. This means that the nitrogen radical density itself is very small when the total gas flow of N_2_/H_2_ is 100 sccm. On the other hand, with increasing the total gas flow rate, the GaN layer becomes flatter and the growth rate is decreased as shown in Figs. 17 and 18.

##### Change of the N_2_/H_2_ total gas flow rate with the same N_2_/H_2_ gas flow ratio of 1:1

**Figure 19:**
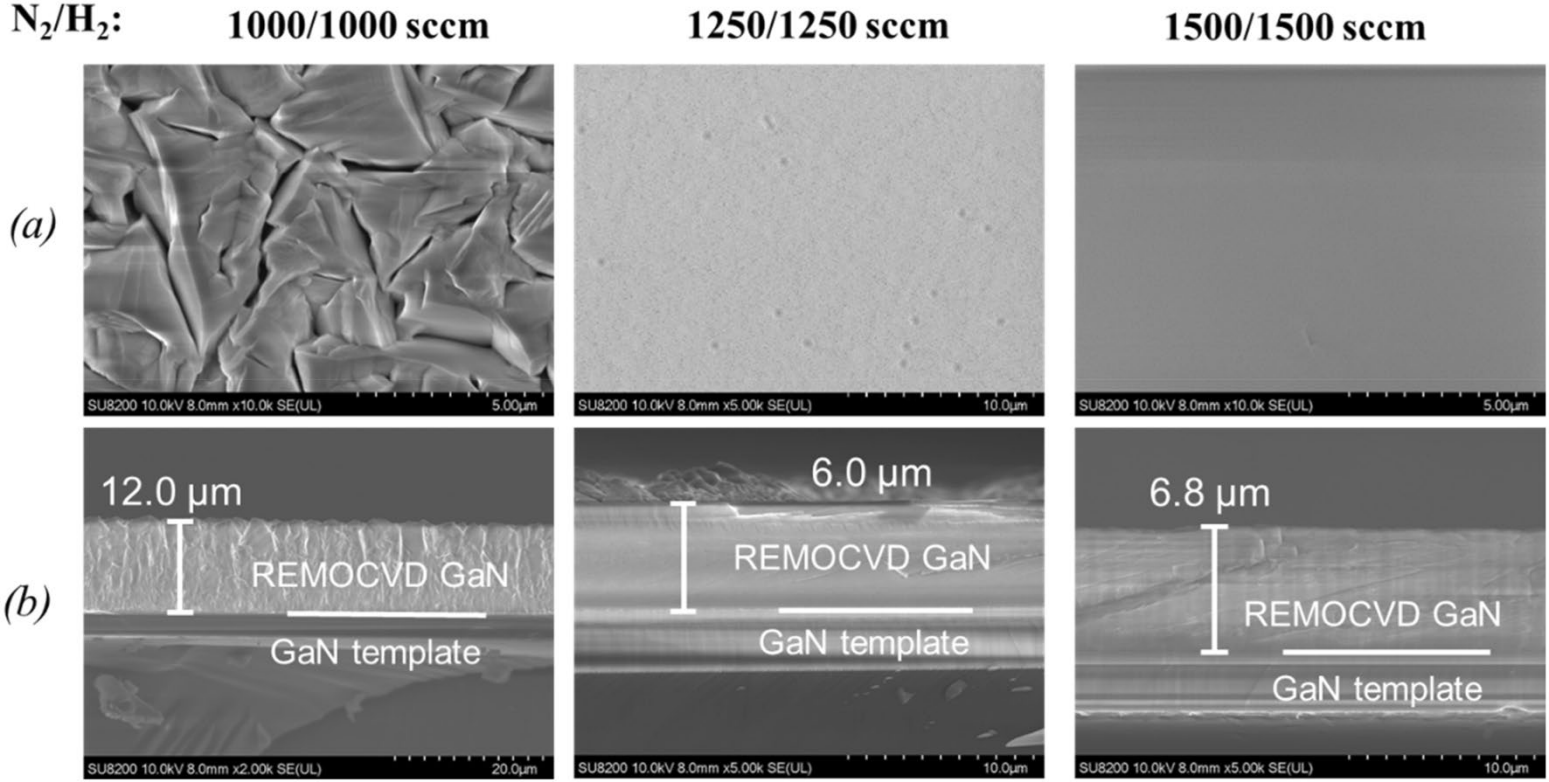
Growth of GaN on GaN templates as a function of the N_2_/H_2_ total gas flow rate with the same N_2_/H_2_ gas flow ratio of 1:1. (**a**) Surface morphology and (**b**) cross-section by SEM. Growth conditions; 800 °C, 120 min, 600 W, 300 Pa, TMG + 5 °C/H_2_ carrier gas 20 sccm.

Since it was found that the density of nitrogen radical species can be increased as shown in Fig. 18 and the growth rate can be made the highest with the N_2_/H_2_ ratio of 1:1 as shown in Fig. 16, we have increased the N_2_/H_2_ total gas flow rate from 2000 to 3000 sccm. It was found that the very flat GaN can be grown with a growth rate of about 3.4 μm/h as shown in Fig. 19.

### Evaluation of crystal quality

As shown above, we have optimized the growth conditions such as plasma power, chamber pressure, total gas flow rate and N_2_/H_2_ flow ratio, and finally could obtain very flat GaN epitaxial growth with a reasonably high growth rate. The optimized growth conditions are as follows.

- Plasma power; 600 W;
- N_2_/H_2_ gas flow rate: N_2_; 1500 sccm, H_2_; 1500 sccm;
- Chamber pressure: 300 Pa;
- TMG flow rate: 2.4 sccm (TMG bottle temperature; + 5 °C with an H_2_ carrier gas of 20 sccm);
- Growth temperature: 800 °C.

**Figure 20:**
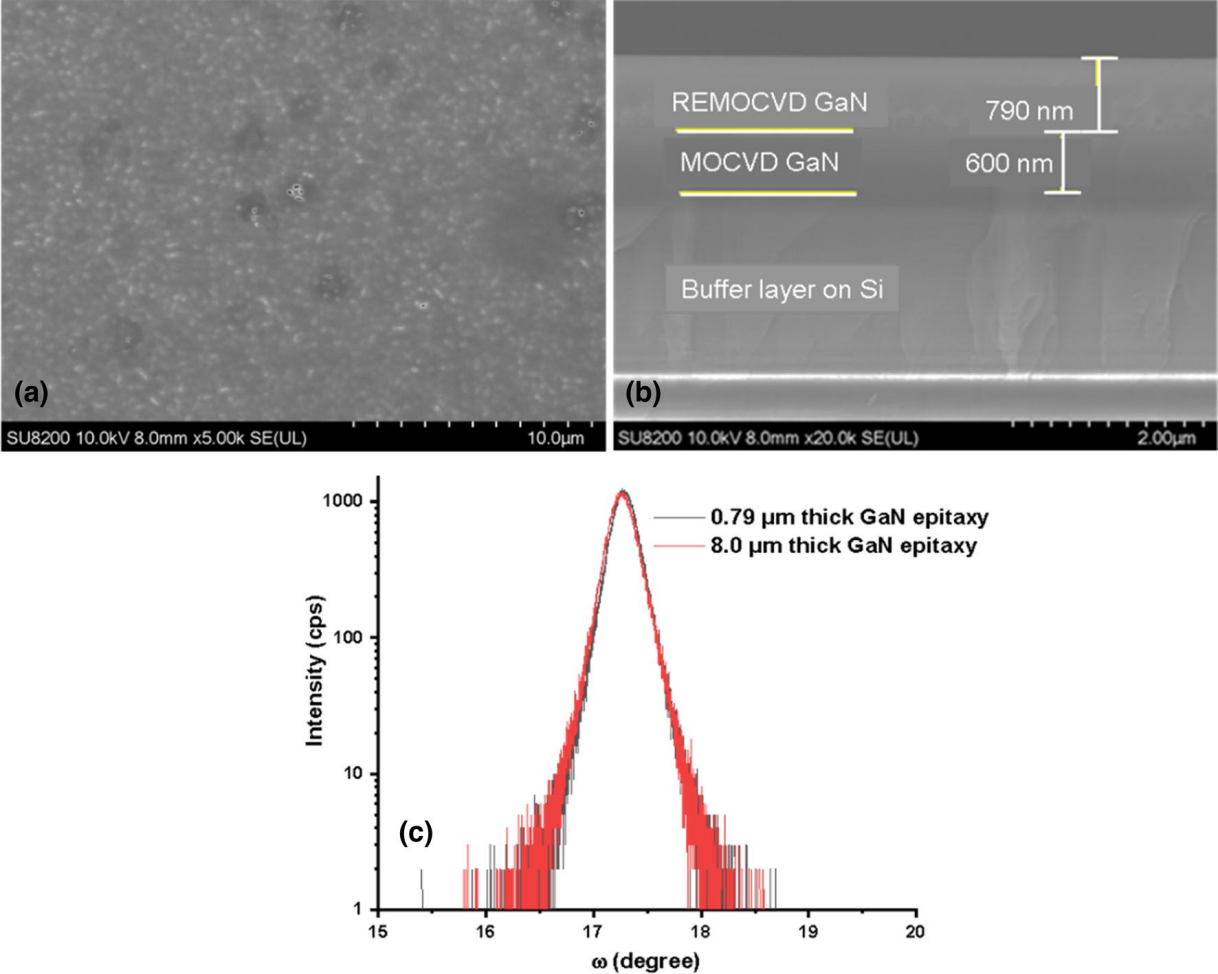
GaN on a GaN template under the best conditions with a growth time of 15 min. (**a**) Surface morphology, (**b**) cross-section by SEM and (**c**) the omega scan of thin and thick GaN. Growth conditions; 800 °C, 600 W, 300 Pa, TMG + 5 °C/H_2_ carrier gas 20 sccm.

**Figure 21:**
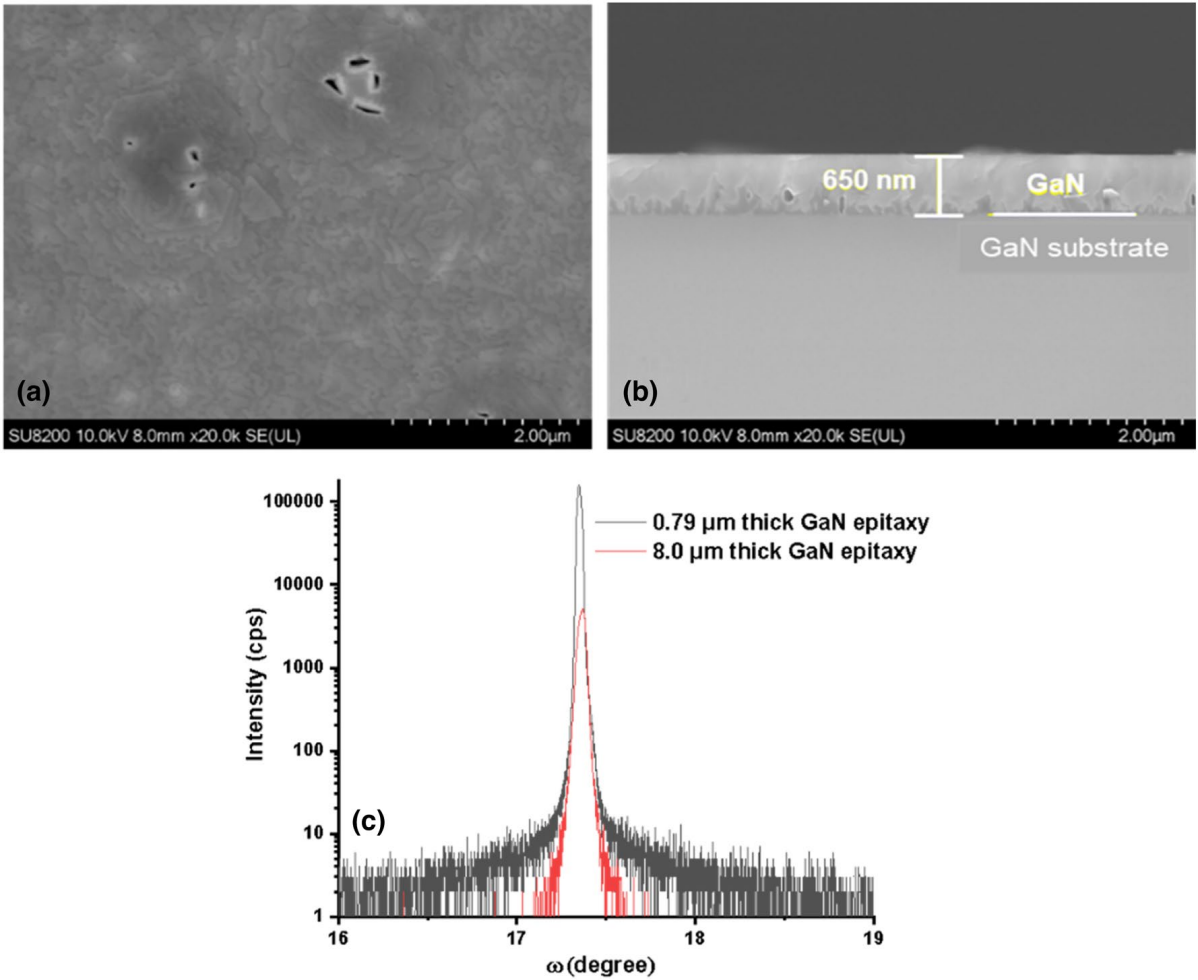
GaN on a bulk GaN substrate under the best conditions with a growth time of 15 min. (**a**) Surface morphology, (**b**) cross-section by SEM and (**c**) the omega scan of thin and thick GaN. Growth conditions; 800 °C, 600 W, 300 Pa, TMG + 5 °C/H_2_ carrier gas 20 sccm.

According to the above conditions we have grown GaN on a GaN/Si template and a bulk GaN substrate as shown in Figs. 20 and 21 respectively. It is clearly shown that very smooth quality GaN could be grown. In the case of GaN grown on the GaN/Si template, XRD-FWHM for the (0002) plane was 977 arcsec and this value is very similar to that of the GaN/Si template. In the case of the bulk GaN substrate, the XRD-FWHM for the (0002) plane was 72 arcsec and this value is very close to that of the bulk GaN substrate. The 8 µm thick GaN films were grown on GaN/Si template and bulk GaN substrates and the results were shown in Figs. 20 and 21. The XRD FWMH for the (0002) plane of thick GaN grown on GaN/Si template and bulk GaN substrates were 875 and 110 arcsec respectively. For the case of GaN grown on the GaN/Si template, the FWHM value is better than the FWHM of the GaN/Si template. Thus we could prove that under the present growth conditions with the pBN inner shield which we have optimized, we could achieve a remarkable crystal quality of GaN at such a low temperature as 800 °C with a high growth rate comparable to conventional MOCVD.

## Discussion

As it was explained in the introduction, we have developed the REMOCVD method which is a very promising technology to replace the MOCVD method. It was shown that the REMOCVD method has the following advantages against the MOCVD method.

1. Nitrides can be grown without ammonia gas. Only from nitrogen and hydrogen gases, we can grow nitrides. This is a big advantage of plasma technology since nitrogen radical species can be generated by plasma only from nitrogen and hydrogen gases as the reaction of N* and H*, the reverse reaction against ammonia gas where nitrogen radical species can be generated thermally from ammonia gas.
2. In the case of MOCVD, nitrogen species are generated thermally and the density is too small so that most of the ammonia gas must be exhausted without being used for the growth of nitrides and is decomposed by expensive detoxication systems and thrown away outside. This increases the production cost of MOCVD and it is known that the 1/3–1/4 production cost is of ammonia gas and detoxification systems. On the other hand, REMOCVD has a good cost performance since only nitrogen and hydrogen gases are necessary.
3. Since nitrogen radical species are generated from nitrogen and hydrogen gases by plasma, it is not necessary to increase the temperature as in the case of MOCVD where nitrogen radical species are generated thermally, and the temperature should be increased higher than 1050 °C. By REMOCVD, it was proved that various nitrides can be grown at much lower temperatures than MOCVD, for instance, we have shown experimentally that GaN can be grown at 800 °C, AlN at 600 °C, AlInN at 600 °C and InN at 200 °C^32–64^.
4. Since the growth temperature is much lower than MOCVD, REMOCVD has a big advantage for heteroepitaxial growth because the difference of lattice constant and thermal expansion becomes smaller at lower temperatures so that cracking or bending of epitaxial wafers can be solved, the big issue of MOCVD where it is very difficult to produce industrially large sides GaN/Si epitaxial wafers, for instance large diameters than 6 in. to 300 mm.
5. Since the growth temperature can be made lower, it becomes easy to grow nitrides with high In content nitrides. This opens many future applications using In contained nitride devices such as long wavelength optical devices. Even the fact that InN can be grown easily is very promising for the application of high mobility InN for high-frequency electronic devices and beyond CMOS planer devices.

**Figure 22:**
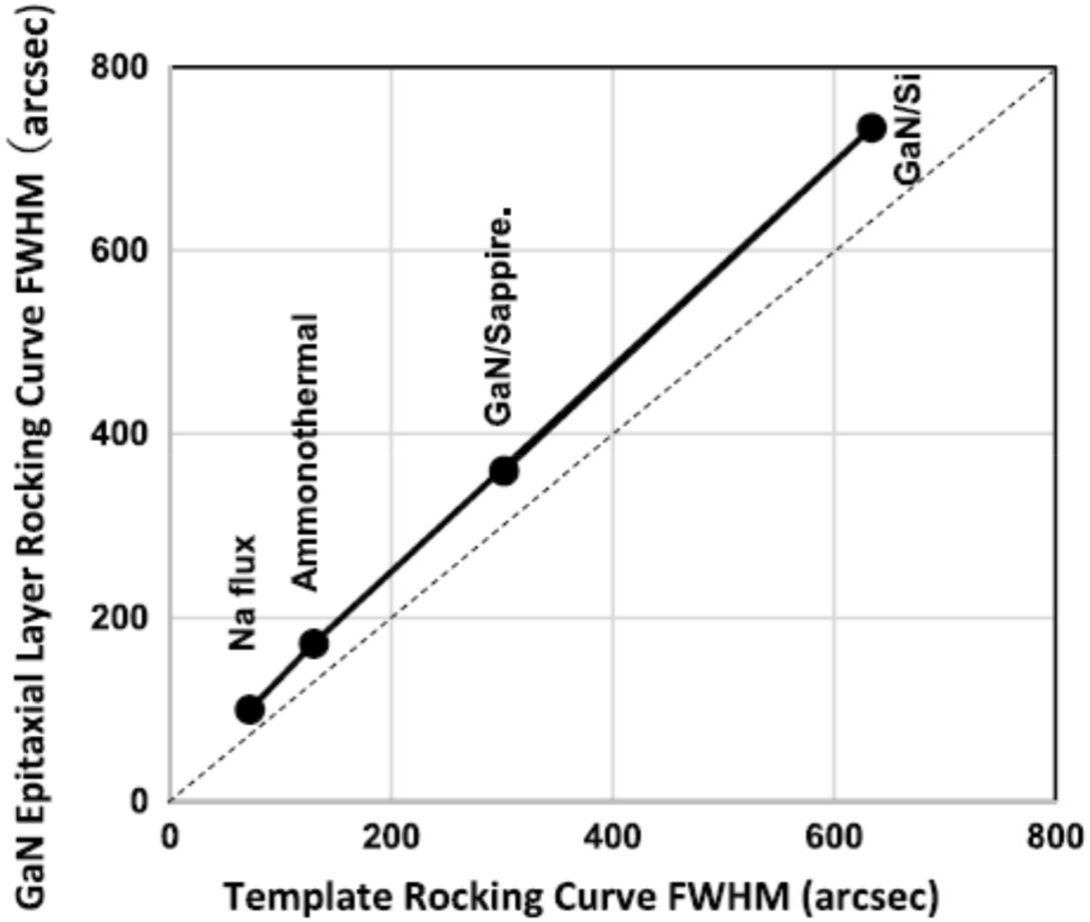
FWHM of epitaxial layers as a function of the substrate FWHM. Reproduced with permission^27^ Copyright 2020, Wiley–VCH GmbH.

There was an argument that low-temperature growth using plasma technology does not allow high crystalline quality nitrides with the belief that individual atoms cannot diffuse on the surface and arrange in a good lattice position to grow appropriately arranged crystal structure. This belief is however against a very important scientific knowledge that the surface diffusion coefficient is increased as a function of T(temperature)/Tm(melting point), which is very well-known experimentally and theoretically^65,66^. Since the melting point of Ga, Al and In is very low after MO gases are decomposed to metallic atoms on the surface, they diffuse very fast even when the temperature is low. It also should be noted that on the substrate, MO gases are decomposed to Ga, Al and In even at low temperatures as measured by spectroscopic ellipsometry^27^. Thus they move to the appropriate growth position such as terraces and when nitrogen radicals come to the terrace and bind with the metallic atoms, nitrides can be grown by the step flow mechanism from the terrace so that even at lower temperatures, high-quality nitrides can be grown. It was shown experimentally for GaN, AlN, AlInN and InN that they could be grown at low temperatures. Figure 22 shows the summary of GaN growth at 800 °C on various templates and substrates. It is known that the GaN crystal quality measured by XRD is very close to that of templates and substrates. It means that we can obtain high-quality nitride epitaxial layers even at low temperatures.

**Figure 23:**
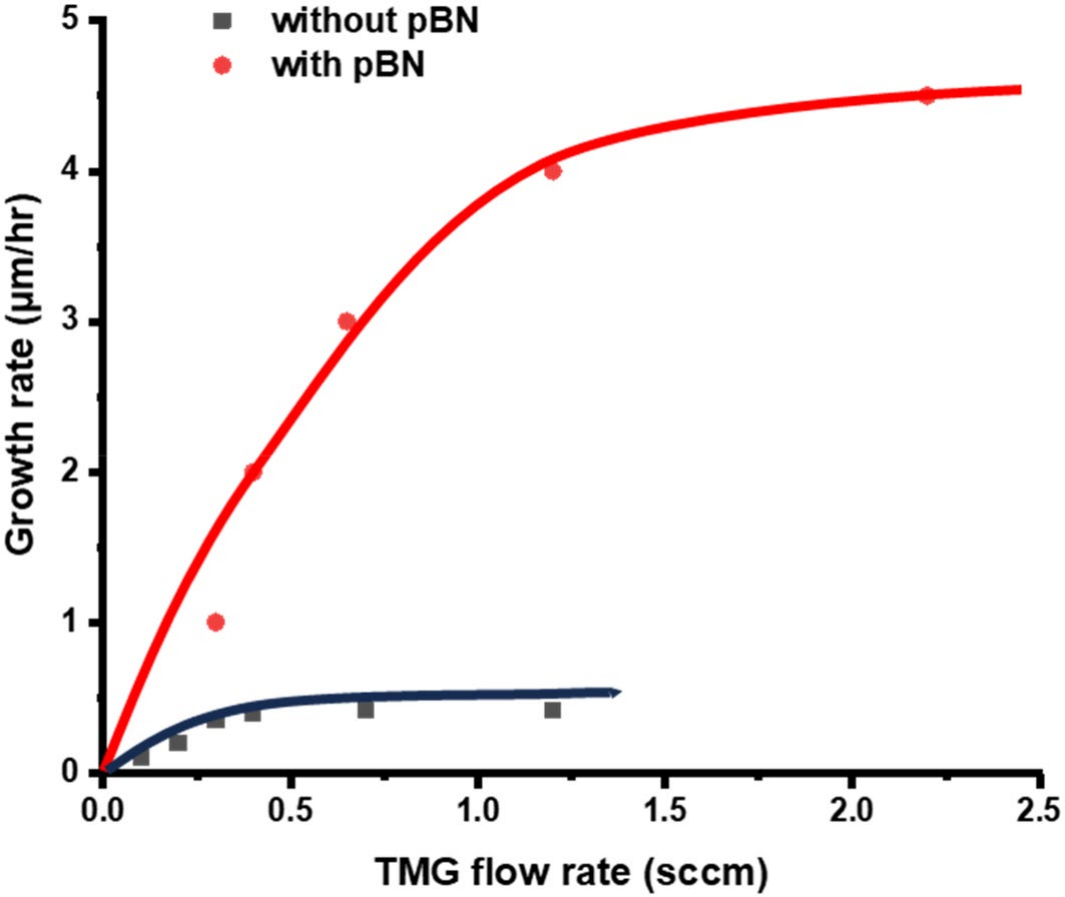
Growth rate of GaN with and without the pBN inner shield. Growth conditions; 800 °C, 300 Pa, 600 W.

The REMOCVD method has however a disadvantage in that the growth rate was very low 0.2–0.8 μm/h which is much lower than those of MOCVD. This is a big issue for REMOCVD to be used as an industrial application. We, however, believed that the growth rate could be increased since it was known and proved that III-group metals diffuse very fast on the growth surface^27,65,66^ and as it is explained in the introduction, the reason why the growth rate is not increased may be due to the deactivation of nitrogen radicals by the collision to the stainless inner wall or due to low electron temperature which cannot increase the number of atomic nitrogen radicals or because the generated radicals are not focused towards the substrate surface. As shown experimentally in this work, we could succeed in growing high-quality GaN even at a low temperature of 800 °C with a growth rate of 3.2 μm/h as shown in Figs. 20 and 21. Depending on the REMOCVD system, we need to optimize the growth conditions but when it is performed, it was shown that the growth rate can be increased as we predicted and it is shown in Fig. 23. Together with the growth rate, we could achieve a remarkably high crystal quality GaN at such a low temperature as 800 °C without toxic ammonia gas.

The present result that showed the high growth rate and high crystal quality of GaN by REMOCVD will open up vertical GaN devices for high-power electronics. We therefore believe that REMOCVD is a very promising nitride growth technology for the future to replace the MOCVD method.

## Conclusion

In the present work, we have shown that the growth rate of GaN by REMOCVD can be increased from 0.4 to 3.3–4.5 μm/h even at low temperatures as 800 °C by installing a pBN inner shield. We believe that the role of the pBN inner shield is to prevent nitrogen radical species from colliding with the inner stainless wall and being deactivated or to increase the electron temperature which can increase the number of atomic nitrogen radicals or focus the generated radicals towards the substrate surface. To grow high quality and flat epitaxial later growth, it was found that the growth conditions such as plasma power, chamber pressure, TMG flow rate, N_2_/H_2_ source gas total flow rate and N_2_/H_2_ gas ratio must be optimized. When these factors are optimized, it was proved that we can grow flat and high-quality GaN. We still do not know how much is the maximum growth rate. We believe that further optimization makes it possible to increase the growth rate further. The REMOCVD method is, therefore, a very promising industrially applicable growth technology in the near future for replacing the MOCVD method, especially for high-power vertical GaN/GaN devices.

## Data Availability

The datasets used and/or analysed during the current study are available from the corresponding author upon reasonable request.
