# Author Correction: Epitaxial growth of high-quality GaN with a high growth rate at low temperatures by radical-enhanced metalorganic chemical vapor deposition

**DOI:** 10.1038/s41598-024-82619-w

**Published:** 2024-12-20

**Authors:** Arun Kumar Dhasiyan, Frank Wilson Amalraj, Swathy Jayaprasad, Naohiro Shimizu, Osamu Oda, Kenji Ishikawa, Masaru Hori

**Affiliations:** Center for Low-Temperature Plasma Science (cLPS), Nagoya University, Furo-cho, Chikusa, Nagoya 464-8603 Japan

Correction to: *Scientific Reports* 10.1038/s41598-024-61501-9, published online 13 May 2024

The original version of this Article contained an error in the legend of Figures 20 and 21, where an inset was mistakenly included.

Figure 20

“GaN on a GaN template under the best conditions with a growth time of 15 min. (a) Surface morphology (inset is the RHEED pattern), (b) cross-section by SEM and (c) the omega scan of thin and thick GaN. Growth conditions; 800 °C, 600 W, 300 Pa, TMG + 5 °C/H_2_ carrier gas 20 sccm.”

now reads,

“GaN on a GaN template under the best conditions with a growth time of 15 min. (a) Surface morphology, (b) cross-section by SEM and (c) the omega scan of thin and thick GaN. Growth conditions; 800 °C, 600 W, 300 Pa, TMG + 5 °C/H_2_ carrier gas 20 sccm.”

And Figure 21

“GaN on a bulk GaN substrate under the best conditions with a growth time of 15 min. (a) Surface morphology (inset is the RHEED pattern), (b) cross-section by SEM and (c) the omega scan of thin and thick GaN. Growth conditions; 800 °C, 600 W, 300 Pa, TMG + 5 °C/H_2_ carrier gas 20 sccm.”

now reads,

“GaN on a bulk GaN substrate under the best conditions with a growth time of 15 min. (a) Surface morphology, (b) cross-section by SEM and (c) the omega scan of thin and thick GaN. Growth conditions; 800 °C, 600 W, 300 Pa, TMG + 5 °C/H_2_ carrier gas 20 sccm.”

The original Article has been corrected.

